# Microbial Enzyme Production Using Lignocellulosic Food Industry Wastes as Feedstock: A Review

**DOI:** 10.3390/bioengineering3040030

**Published:** 2016-11-16

**Authors:** Rajeev Ravindran, Amit K. Jaiswal

**Affiliations:** School of Food Science and Environmental Health, College of Sciences and Health, Dublin Institute of Technology, Cathal Brugha Street, Dublin D01 HV58, Ireland; rajeev.ravindran@mydit.ie

**Keywords:** food industry waste, enzyme production, fermentation strategies, enzyme purification, lignocellulose

## Abstract

Enzymes are of great importance in the industry due to their substrate and product specificity, moderate reaction conditions, minimal by-product formation and high yield. They are important ingredients in several products and production processes. Up to 30% of the total production cost of enzymes is attributed to the raw materials costs. The food industry expels copious amounts of processing waste annually, which is mostly lignocellulosic in nature. Upon proper treatment, lignocellulose can replace conventional carbon sources in media preparations for industrial microbial processes, such as enzyme production. However, wild strains of microorganisms that produce industrially important enzymes show low yield and cannot thrive on artificial substrates. The application of recombinant DNA technology and metabolic engineering has enabled researchers to develop superior strains that can not only withstand harsh environmental conditions within a bioreactor but also ensure timely delivery of optimal results. This article gives an overview of the current complications encountered in enzyme production and how accumulating food processing waste can emerge as an environment-friendly and economically feasible solution for a choice of raw material. It also substantiates the latest techniques that have emerged in enzyme purification and recovery over the past four years.

## 1. Introduction

**Figure 1:**
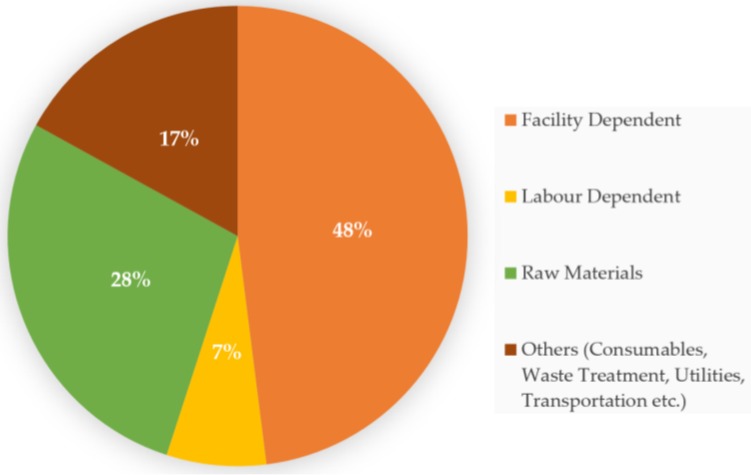
Breakdown of annual operating cost of a typical enzyme production plant [2].

Enzymes are biological catalysts found in all living systems. Enzymes are proteinaceous in nature and catalyse a variety of reactions. For centuries enzymes have involuntarily been used in the form of bacterial or plant extracts for making wine, cheese, bread, beer and vinegar, and for manufacturing commodities such as leather and linen. However, it has only been a few decades since purified enzymes have found extensive application in manufacturing processes [1]. A major issue with the application of enzymes in industrial processes is the cost associated with it. Large-scale enzyme production is a capital-intensive process and the application of enzymes in different manufacturing processes indirectly influences the cost of the finished product. Much of the annual operating cost of an enzyme production facility is attributed to plant equipment and installation (Figure 1). However, 28% of the operating cost is contributed by raw materials [2].

Lignocellulose is a great source of cheap carbohydrates and thus has been used over the past decades as a raw material for the production of a range of high value products, such as bioethanol, organic acids, enzymes and biodegradable plastics [3]. Lignocellulose is made up of lignin and carbohydrate polymers, like cellulose and hemicellulose, along with pectin and traces of salts, minerals and ash [4]. Using food material for valorization has sparked a worldwide debate, encouraging scientists to look for other feasible alternatives [3]. Most of the processing waste generated by the food industry is inedible and lignocellulosic in nature [5]. Due to its complex structure, lignocellulose is highly recalcitrant and cannot be directly used for microbial processes. However, subjecting lignocellulose to pretreatments and enzymatic hydrolysis releases fermentable sugars, which can utilized by enzyme-producing microbes for their growth and sustenance [6]. This article reviews the latest technology involved in the production of cost effective, efficient enzymes by the valorization of food industry waste.

## 2. Market Potential

The global enzyme industry is growing at a fast pace. It was worth almost $4.8 billion in 2013 and is estimated that by 2018 will be worth a staggering $7.1 billion [7]. Enzymes are predominantly used for the production of several products that we use in our day-to-day lives [8]. Besides, new-found interest in bioenergy has led to an increased demand for enzymes applicable in the biofuel sector [9]. Enzymes can be prepared for customized applications for different industrial processes with the help of recombinant DNA technology and protein engineering. According to a report published by the “National Renewable Energy Laboratory”, cellulosic ethanol prices are highly dependent on the cost of saccharification enzymes that can break down complex carbohydrates into fermentable sugars. Therefore, decreasing the cost of enzymes can increase the market potential of biofuels and also other value-added products [10].

## 3. Types of Food Waste

The central dogma of utilization of waste for product development is the stability, safety and economic feasibility of product/process development. Apart from the high carbohydrate content, the additional nutrients found in food industry wastes make them ideal media components for microbial growth. Food processing waste can be categorized into six types, based on its source of origin: (i) vegetable trimmings and pulp; (ii) starch-based waste; (iii) fruit peels and pulp; (iv) spent grains/vegetable oil cakes; (v) meat and fish waste and (vi) dairy waste. Meat, fish and dairy waste are outside the scope of this article. All the other categories are rich in carbohydrates and differ from one another with respect to factors such as structure, chemical composition, moisture content, etc. While the majority of the composition is dominated by non-starch carbohydrates and lignin, grain waste varieties also contain high amounts of proteins, lipids, starch and glucans. The exact percentage composition of grain varieties differs according to the season of harvest [11,12,13]. Apart from polysaccharides, proteins and lipids food industry wastes, such as apple pomace, are also a major source of dietary fibre, polyphenols and bio-active compounds [14,15]. The diversity of compounds found in food industry waste can result in some of them acting as growth enhancers for microbial processes. Banana and plantain pulp extract was found to promote the growth of gram-positive bacteria [16].

## 4. Economic Impact of Food Waste Valorization

The valorization of food processing waste has become a topic of debate in the current scenario due to the emphasis of the EU on “Bioeconomy”. Bioeconomy is a concept introduced by the European Commission in 2012, which includes conversion of food processing waste into value-added products [3]. This is backed by initiatives by the European Union to tackle food waste thorough legislation and reduce the amount of food waste sent to landfill from 40 metric tonnes to 4 metric tonnes by 2020. The European Commission adopted a “circular economic package” in 2015, which was comprised of various proposals for legislations, with the aim to boost global competitiveness, foster economically sustainable growth and to create new jobs. Through this endeavor, the commission seeks to “close the loop” of product lifecycles by increased recycling and reuse, thereby contributing to the environment and economy. One of the major features of this measure is to promote re-use and encourage industrial symbiosis by turning one industry’s waste into another industry’s raw material. By achieving this feat, the commission targets reducing material input by 17%–24%, saving €630 billion and reducing total greenhouse gas emissions [17].

## 5. Food Industry Waste (Global Status)

Food waste is generated from the non-products flow of raw materials, whose collection and processing for reuse cost more than their economic value, and are thus discarded as waste. An estimated 89 million tonnes of food waste is produced every year in the European Union (EU)-27, with the manufacturing sector contributing to over 38% [18]. Waste produced by the agriculture and manufacturing sector is in a concentrated manner, which is easier to collect, distribute and valorise to form value-added products. Steps taken by the industry for higher sustainability, processing efficiency and improving green credentials have led to the development of innovative strategies for the valorization of food waste [19]. Considerable efforts are being made by the European Union, with the implementation of strict laws and high cost associated with the disposal of food waste as landfill, which encourages its application for the production of value-added products [3,18].

Most wastes generated from food processing industries are lignocellulosic in nature. Cellulose is a major polysaccharide found in lignocellulose and is made up of repeating glucose units. Besides cellulose, plant-based food wastes are rich in pectin, inulin, xylan, mannan, glucan, starch, etc., depending upon the nature of the waste product. Being rich in polysaccharides, lignocellulosic waste material is an obvious choice of raw material for the production of lignocellulose-degrading enzymes. Microorganisms produce enzymes that are specific to each polysaccharide component for the release of sugars that can be metabolized for growth energy and cell maintenance. Since they are a cheap source of carbohydrate, the efficiency of using lignocellulose as a raw material for industrial microbial processes is being extensively researched [20]. Lignocellulosic food waste has been studied as a potential media component for the production of various industrially important enzymes—cellulose-degrading enzymes, in particular, because of the abundance of cellulose in it. Several studies have reported the use of various lignocellulosic substrates. One of the most important applications of the utilization of lignocellulosic waste is for the production of lignocellulose-degrading enzymes viz. cellulase, hemicellulase and laccase. Laccase enzyme degrades lignin and is widely used in the textile, paper and pulp and petroleum industries, as well as in bioremediation [21]. Cellulase and hemicellulase, on the other hand, find applications in production of second-generation biofuels, bioethanol in particular. The on-site production of cellulase, coupled with the hydrolysis of biomass on the same location, is an important strategy for cost reduction in the development of sustainable ethanol production [22]. Table 1 summarizes the lignocellulosic sources that have been used as raw material for enzyme production. However, lignocellulosic substrates need to undergo certain upstream processes before they can be used as substrate for production processes. Structural constraints and component fractions present in lignocellulose do not support the prolific growth of microorganisms in order to produce the desired product via fermentation. The next section discusses the factors that influence the use of lignocellulose as a suitable raw material for enzyme production.

## 6. Challenges in Enzyme Production Using Lignocellulosic Food Waste

### 6.1. Lignocellulose as a Raw Material

Lignocellulose is essentially a complex polymer which is made up of polysaccharides and phenolic polymers (lignin). All plant matter is composed of lignocellulose, and lignin is the most recalcitrant substance found in them. It imparts physical strength to the plant cell wall. It is a complex polymer made up of coniferyl alcohol, synapyl alcohol and *p*-hydroxyphenyl alcohol [23]. The presence of lignin in lignocellulose prevents its effective enzymatic degradation and subsequent utilization of fermentable sugars by microbes [24]. This calls for effective measures for lignin removal through various pretreatment techniques [25,26]. The crystalline nature of lignocellulose is another major hurdle in its efficient utilization. Crystallinity of the biomass material is imparted by the presence of crystalline cellulose, which, if not converted into its amorphous form, is not susceptible to enzymatic hydrolysis [27,28].

Cellulose and hemicellulose are two components in the plant cell wall, which, when hydrolyzed, result in the release of fermentable sugars (glucose, xylose, galactose, etc.) [29]. Enzyme-producing microbes depend on glucose as their main carbon source. High cellulose content is therefore desired in a potential lignocellulosic substrate. In such cases, the presence of hemicellulose can interfere with cellulose breakdown, glucose formation and uptake [30]. However, the production of different enzymes is susceptible to the presence of specific substrates in the media composition. For example, the presence of xylans and arabinoxylans in substrates has been reported to boost xylanase production [31]. Also, uncommon sugars, such as fructooligosaccharides and inulin based fructans, facilitate better inulin production by several microbial species [32]. The availability of the lignocellulosic substrate and its cost are also important aspects in the choice of substrate.

### 6.2. Pretreatment of Lignocellulose

The utilization of cellulose in biomass as a carbon source involves the adsorption of enzymes on the substrate surface, the synergistic effects of other protein components on hydrolysis, and the release of hydrolyzed product into the bulk liquid. Along with lignin and hemicellulose, pectin and acetyl groups are some of the compositional factors that influence lignocellulose degradation [33]. The removal of pectin by the enzymatic treatment of lignocellulose with the pectinase enzyme along with cellulolytic enzymes has a positive effect on biomass utilization [34]. Degree of polymerization, plant protein–enzyme interaction, structural rigidity, accessible surface area for enzymatic degradation and porosity and the residual surface area of biomass also have a pronounced effect on substrate utilization and assimilation [35]. Pretreatments are necessary to address these issues by bringing in structural and compositional changes in lignocellulose. Pretreatment strategies employ high pressure or temperature, or a combination, for the reduction of recalcitrant components from lignocellulose. Sophisticated disruption techniques, such as ultrasonication, microwave exposure, treatment with corrosive liquids, such as acids and alkali, and enzymatic hydrolysis can also be part of effective pretreatment measures [36]. However, pretreatment techniques involving heat and acid hydrolysis give rise to enzyme and microbial growth inhibitors that can impede the production process [37,38].

### 6.3. Choice of Microorganism

Considerable research has been conducted to reveal several microorganisms belonging to the fungi, yeast, bacteria and actinomycetes categories. Screening of microorganisms is one of the most efficient means of finding new enzymes viable to the industry. This is particularly true in the case of thermophilic microorganisms that are isolated from exotic locations and subsequent extraction of enzymes. One of the greatest advantages of employing thermophilic microorganisms for enzyme production is reducing the risk of contamination due to bioprocessing operations being conducted at higher temperatures. Furthermore, elevated temperatures also result in lesser viscosity and greater solubility of substrates, subsequently resulting in increased product yields due to favourable displacement of the equilibrium in endothermic reactions [39].

Enzymes of microbial origin employed in the industry are commercially available as enzyme preparations. These preparations not only contain the desired enzyme, but also other metabolites of the production strain, along with additional preservatives and stabilizers that are food grade and comply with applicable regulatory standards. While evaluating the safety of an enzyme, the safety of the production strain remains the primary consideration. Toxigenic potential is defined as the ability of a microorganism to produce chemicals (toxins) that can cause food poisoning [40]. Strains that are meticulously characterized to be non-pathogenic and non-toxigenic, particularly those with a history of being safe, are reasonable choices for the production of industrial enzymes [41]. A majority of the industrially important enzymes has been derived from a rather small group of bacterial and fungal strains, primarily *Bacillus subtilis*, *Bacillus licheniformis*, *Aspergillus niger* and *Aspergillus oryzea*. These microbes have historically been used for the commercial production of various metabolites, leading to a thorough understanding of their characteristics and metabolic reactions, and have been documented to be efficient for industrial scale production. They can also be genetically manipulated easily and are known for their ability to overexpress proteins of interest in fermentation media. These features make these microorganisms extremely desirable as hosts for a variety of heterologous enzymes. Furthermore, genetic engineering has enabled several microorganisms with no history of use in the industrial production of native enzymes, such as *Escherichia coli* K-12, *Fusarium venenatum* and *Pseudomonas fluorescens* to be successfully utilized as hosts for expression of industrially important enzymes [42].

Wild-type strains produce a variety of extracellular enzymes, which may naturally produce enzymes that have industrial importance. A common method exploited to find these microbes is bioprospecting. Microbes are collected from specific environmental niches and their ability to hydrolyze specific substrates is investigated. Subsequently, the best candidate is selected based on screening for the production of an enzyme of interest. Another method is analyzing the genetic composition using metagenomic tools. Probes and group-specific primers are employed to find new enzymes. The major drawback with this method is qualitative: the metabolic potential cannot be measured since the isolation and culturing of the microorganism is not performed. Comparative genome analysis of microbial strains assists in the screening of prospective microbes in a short time. This facilitates the evaluation of the proteome of the microorganism [43].

The concept of metabolic engineering was introduced by Bailey in 1991 and relates to ‘the improvement of cellular activities by manipulation of enzymatic, transport, and regulatory functions of the cell with the use of recombinant DNA technology [44]. A metabolic pathway (or pathways) associated with the production a desired chemical compound by fermentation is/are over-expressed in a cell by genetic engineering employing classical mutagenesis and selection and/or recombinant techniques. Cells are “evolved” in the laboratory to make them tolerant to high product concentrations by removing the normal genetic and biochemical regulation of the genes and enzymes associated with the pathway by genetic manipulation. Finally, a robust fermentation process is developed that allows mass production of the desired compound [45].

While traditional metabolic pathway engineering approaches have been successful in producing engineered microbes, novel techniques need to be called upon to develop microorganism and processes that are cost-competitive with existing large-scale, low-cost chemical manufacturing processes, to produce the same compounds in high-volume and at low-cost. Techniques, like genome sequencing, and new fields of study, like bioinformatics, systems biology and metabolomics, have greatly helped researchers to embellish the competences of metabolic engineering over the past decades to deliver new, highly engineered organisms that are capable of high throughput performance using renewable resources as substrates, lowering the cost of production even further [46,47].

### 6.4. Fermentation Strategies

#### 6.4.1. Solid State Fermentation

**Figure 2:**
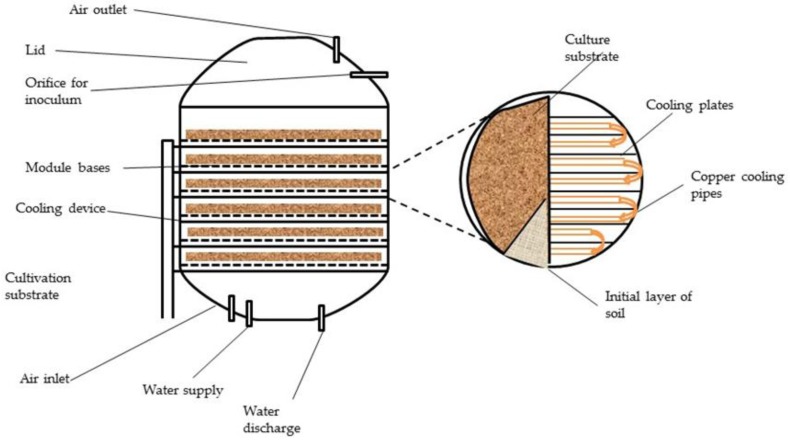
Schematic of solid state fermenter for conversion of lignocellulosic biomass to enzymes.

Solid state fermentation has been used for the production of several industrial enzymes. This mode of fermentation encourages the growth of fungal species, such as ascomycetes, basidiomycetes and deuteromycetes, on solid substrates, and the development of conidiospores in particular [48]. Most of the industrial enzymes are of fungal origin. The genetic expression of fungal organisms differs in solid state fermentation and submerged fermentation [49]. However, solid state fermentation has not been adopted for large-scale production because of its inability to standardize processes and limited reproducibility of results. Temperatures can rise to unprecedented levels with fewer measures to control them, which can denature the enzymes that are produced in the reactor. Aeration has been proposed as a measure to address this issue. This can, however, lead to water loss due to evaporation [50]. Figure 2 depicts a general schematic of a large-scale solid state fermentation unit.

Advances in fermentation technology have seen some success in formulating methods to make solid state fermentation an amenable process. Ito et al. [51] constructed a non-air flow box (NAB) with a moisture permeable flouropermeable membrane. Water vapour escapes through the membrane keeping the substrate dry, resulting in uniform culture growth and rapid enzyme production with high reproducibility [52]. Solid state fermentation promises high volumetric productivity with a high concentration of products, along with lesser effluent production, leading to minimal downstream processing [53]. However, a study involving the use of organic waste for the production of enzymes using the solid state fermentation regime reported the emission of volatile organic compounds (VOC) such as CH_4_, N_2_O and NH_3_ [54].

#### 6.4.2. Submerged Fermentation

**Figure 3:**
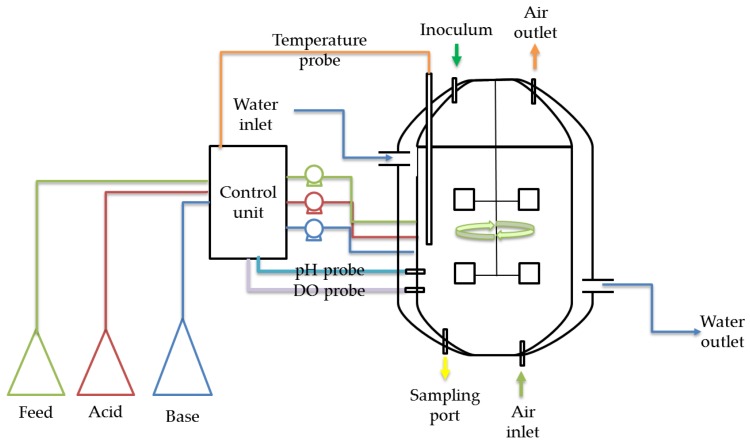
Schematic of submerged fermenter (stirred tank reactor) for conversion of lignocellulosic biomass to enzymes.

The submerged fermentation strategy is the most sought-out method for large-scale production of enzymes. Figure 3 represents a stirred tank reactor, which is one of the modes of submerged fermentations used for enzyme production using lignocellulose as substrate. It involves water-based medium within the reactor, which helps in maintaining the pH and temperature and provides provisions for aeration and agitation within the vessel. A sterile environment can be maintained within the reactor with lesser chance of contamination. Submerged fermentation maintains a homogenous environment, with the reactor facilitating better control over process parameters. While this helps optimization studies and the even distribution of nutrients and oxygen to the growing microbe, the presence of negative factors such as butylated hydroxytoluene, hydrogen peroxide and metal ions, which induce oxidative stress, can undermine the whole operation [55]. Several filamentous fungal species are employed for the production of various enzymes and bioactive compounds. Although submerged fermentation is employed for the cultivation of these microbes in the industry, several studies prove that it is not the best method available [56]. Submerged fermentation is not as economical as solid state fermentation due to the requirement of large and sophisticated equipment. A comparative study involving the production of biodiesel revealed that the capital investment required for submerged fermentation was 78% more than that of the solid state mode [57].

#### 6.4.3. Alternative Fermentation Modes

Enzyme production from lignocellulosic substrates essentially involves biomass pretreatment, followed by employing microorganisms specific to the enzyme that needs to be produced. In some cases, enzymatic hydrolysis of pretreated biomass is a key step in this process, which results in a hydrolysate with fermentable sugars. This hydrolysate can then be used as an additive, along with other media components, for enzyme production. Hydrolysis of lignocellulose either involves concentrated acid treatment or a cocktail of enzymes (cellulase and hemicellulase), which results in the release of two main reducing sugars: glucose and xylose. Other sugars that are released include arabinose, mannose and galactose. Rajagopalan and Krishnan [58] employed this strategy to produce α-amylase, using sugar cane bagasse hydrolysate. However, the inclusion of the enzymatic hydrolysis step depends upon the nature of the microbial digestion of the biomass. Certain microbes produce enzymes as a means of breaking down the polymeric structure of lignocellulose. In such cases, the predigestion of lignocellulose using enzymes is an unnecessary step, making the production process economically impractical.

Sequential solid state and submerged fermentation processes have gained popularity among researchers in recent years. This strategy involves a solid state pre-culture fermentation where lignocellulosic biomass is used as substrate. The biomass is enriched with nutrient broth to adjust the moisture content to 70%. Spores are then added in specific ratios with respect to the solid biomass content. The solids state fermentation is performed for 24 h after the fermentation medium is enriched with larger volumes of nutrient medium containing additional amounts of glucose, and rest of the process is performed in submerged conditions. This strategy was reported by [59] while employing *Aspergillus niger* for the production of cellulase, using sugarcane bagasse as substrate.

## 7. Isolation, Purification and Recovery of Enzymes

Once the production stage ends, enzymes are formed in the media in the form of crude extract. Although the crude extract exhibits the desired enzyme activity, low rates of reaction and the presence of impurities, such as media components used for microbial cultivation, metabolites and toxins released by the microorganisms, prevent its use as a commercial entity. Therefore, isolation and purification are the last steps of any enzyme production process. Purifying the enzyme increases its specific activity and removes any unwanted factors from the finished product. Protein purification techniques have been an area of research for many decades owing to the product’s commercial importance. Obtaining the finalized product requires strenuous measures, which start with cell disruption (depending on the source), extraction, fractionation and the final product.

### 7.1. Source of Enzyme

All biological systems are a source of enzymes and there is bound to be considerable variation in the concentration of the concerned enzyme, its activity, stability, availability and presence of inhibitory factors. Traditional animal, plant and microbial sources have given way to genetically-engineered organisms with the introduction of recombinant DNA technology. Eukaryotic proteins cloned and expressed in bacterial hosts, such as *Escherichia coli* and *Bacillus subtilis*, may be located in different locations within the cell (cytoplasm, periplasmic space) or may be truly extracellular. Enzymes accumulating in the periplasm may be released into the fermentation media by changing the culture conditions [60]. However, recombination techniques allow the gene of interest to be equipped with an “affinity tag”, such as His-tag, which will help in the purification of the enzyme. This tag can later be removed by using highly-specific proteolytic enzymes [61].

### 7.2. Isolation of Enzymes

Isolating the protein from a solid source is a compromise between quality and quantity. The best isolation measure should facilitate the release of the enzyme of interest while leaving behind tough contaminants (nucleic acids, bacteria and viruses). Care should also be taken whereby the protein extracted is not degraded/denatured during the process. Homogenization is the most popular method for protein extraction from the cellular environment. Another method used for cell disruption is ultrasonication. Ultrasonication facilitates the disruption of cells and exposes internal proteins to the growth medium. Ultrasonication techniques use high-frequency waves to cause cavitation on the microbial cell wall, thereby destroying it. However, prolonged exposure to ultrasound can denature the protein released upon cell lysis. Therefore, sonication cycles should be optimized in a manner where only cell disruption is achieved while the protein of interest is left intact [62]. The cell disruption technique is usually followed by centrifugation or filtration aimed at the clarification of the extract prior to column chromatography.

Characteristics of the isolation medium are determined by the conditions that are necessary for the stability of the protein released. The main factors that govern the preparation of the isolation medium are pH, detergents, reducing agents, chelators or metal ions, proteolytic inhibitors and bacterial contamination. The pH is usually chosen to be the value in which the enzyme exhibits maximum activity. However, this may not always be the case. In the case of trypsin, maximum activity is attained at pH 8–9, while the enzyme is most stable at pH 3 [63]. Detergents are used to relieve the enzyme of bonds to membranes by hydrophobic interactions. Several of the detergents used for isolation (such as Triton X-100 and Sodium dodecyl sulfate, SDS) do not denature the globular proteins or affect their catalytic activity. The use of detergents is usually limited to the isolation medium. Detergents, being amphiphatic molecules, aggregate to form ‘micelles’ at the critical micelle concentration (CMC). This can interfere with the purification process during column chromatography. Therefore, the concentration of detergent used during isolation must be lesser than CMC [64].

Many enzymes have exposed thiol groups which can oxidize when the protein is released from the cytosol to the growth medium during isolation. This is prevented by the addition of reducing agents such as mercaptoethanol, dithiothreitol (DTT), or ascorbic acid. The concentration of these reducing agents can normally be kept as low as 10–25 mM, while keeping the internal disulphide bonds intact [65]. Metal ions, proteases and bacterial contamination are three problems faced during enzyme isolation. The presence of metal ions leads to the enhanced oxidation of thiol groups and may form complexes with specific groups, which can cause problems. Heavy metals can be removed by treatment with chelating agents such as ethylenediaminetetraacetic acid (EDTA) and ethylene glycol tetraacetic acid (EGTA). EDTA is a buffer whose addition can change the pH of the buffer. Therefore, care should be taken to adjust the pH post addition of EDTA [66].

Proteases that are naturally present in the cell lysate pose a serious threat to the protein of interest. The simplest way to prevent the proteolytic action is by adding protease inhibitors. This can be a bit of a problem in large-scale processes, since proteolytic inhibitors are expensive. Nonetheless, other pragmatic measures to tackle this problem include the adsorption of proteases onto hydrophobic adsorbents and adjusting the pH to a value where proteases are rendered ineffective. Researchers have recently established that the addition of doxycycline indirectly inhibits proteolytic activity of tryptic peptidases [67]. The key to avoiding bacterial growth in enzyme preparation is following measures to ensure sterility. Some buffers, such as phosphate and acetate, among others, are more prone to supporting the growth of bacteria at neutral pH. The addition of antimicrobial agents to buffers whenever feasible is also a tactic to prevent contamination [68].

### 7.3. Fractionation

Fractionation techniques began with the adsorption and precipitation methods. This has now given way to electrophoresis and chromatographic techniques. Each protein is unique in its properties and specific purification protocols must be designed. Every detail pertaining to the protein of interest, such as its characteristics and the nature of its impurities, must be collected and analyzed. Nonetheless, protein purification protocols must be followed in an order, one after the other. This section is dedicated to the recent techniques and development in protein purification.

#### 7.3.1. Precipitation, Centrifugation and Ultrafiltration

Clarification of the cell lysate to remove all cell debris and other particles is achieved by centrifugation at high speeds. This is achievable at laboratory scale, where crude cell preparations can be attained at high speeds of 40,000 g to 500,000 g under refrigerated conditions. In order to complement centrifugation, microfiltration and ultrafiltration have emerged as advancements in filtration techniques to remove contaminating insolubles. Ultrafiltration is a protein separation technique that can separate protein fractions according to their molecular sizes (ranging from >1 to 300 kDa). This method is fast, reliable and inexpensive, while separating salts and other small molecules from protein fractions of larger molecular weight [69].

Precipitation of a desired protein is achieved by the addition of salts, organic solvents, or polymers, or by varying the pH or temperature of the solution. Antichaotropic salts are the most widely used salting out agents. They bind to water molecules, thereby increasing the hydrophobic effect, leading to the aggregation of protein molecules. The most common antichaotropic salt used for protein precipitation is ammonium sulphate [70]. Organic solvents, such as ethanol and acetone, are also used for protein precipitation. Organic polymers function the same way as organic solvents. Polyethylene glycol (PEG) is the most widely used organic polymer for this purpose. The solution, being viscous at high concentrations, can be diluted with buffer. PEG, being uncharged, can be used directly for ion exchange chromatography, to separate proteins [71].

#### 7.3.2. Liquid–Liquid Extraction

Liquid–liquid extraction, also known as aqueous two-phase extraction, is a different way of separating proteins. The experimental setup for liquid–liquid extraction usually consists of PEG as one phase and another polymer, such as dextran, or some salt, as the other. The protein of interest normally migrates to the upper phase (PEG) under favourable conditions, while the contaminating elements, such as other proteins and other particles, accumulate in the lower phase. These can then be removed via centrifugation [72]. Aqueous two-phase systems have been used to extract enzymes from pineapple. Researchers used the polyethylene glycol/potassium phosphate system (18% PEG 1500 molecular weight, 14% phosphate) for the separation of bromelain and polyphenol oxidase. An activity increase of 228% was reported for bromelain with a 4.0-fold increase in purity, while the extracted polyphenol oxidase exhibited 90% activity, with an increased purity of 2.7-fold [73].

#### 7.3.3. Chromatography

Chromatography is a separation technique where two parties are involved: a stationary phase and a mobile phase. As far as protein purification is concerned, the stationary phase is usually a solid substance designed in the form of a column. The mobile phase is usually liquid, such as a buffer of certain pH or salt solutions. Some of the most popular chromatography methods used for protein purification include size exclusion chromatography, ion exchange chromatography, hydrophobic interaction, immobilized metal ion affinity chromatography and affinity chromatography [74].

Size exclusion chromatography separates protein molecules based on their hydrodynamic volume. Size exclusion chromatography usually contains porous beads with different pore sizes unique to each column. Particles in the mobile phase passing through the column will have different hydrodynamic volumes. Those with lesser volume will equilibrate the column faster, thereby eluding at a higher rate. The bigger particles, however, will require more time to travel the entire length of the column. This leads to separation of particles within the mobile phase. Starch and dextran used to be the materials of construction for a size exclusion column. This has now given way to substances such as S-200 Sephacryl beads [75].

Ion exchange (IE) chromatography is a standard technique used for protein purification. The stationary phase used in IE chromatography has charged ligands that get involved in an electrostatic interaction with biomolecules in the mobile phase. The strength of the interaction between protein molecules and the IE column is based on the Z number, which is an indication of the number of binding sites on the protein with respect to ligands [76]. The mobile phase is maintained at a low–medium ionic strength in the beginning of the separation run. During this period, molecules interact with the charged ligands in the stationary phase. The strength of the interaction is determined by the charge location and density on the molecule and ligand. As the salt concentration is increased, the molecules with weak interaction are eluted first, while molecules involved in stronger interactions are eluted later. The stationary phase is made up of resins, which can either be cationic or anionic in nature. Potassium hydroxide is the most popular mobile phase used for IE chromatography [77].

Most protein molecules have hydrophobic areas on their surfaces. In aqueous mobile phases, these molecules form hydrophobic cavities. Lyotropic salts are a group of salts that form liquid crystal upon interaction with an aqueous medium (examples include salts with SO_4_^2−^, Cl^−^, NO^3−^, SCN^−^ and K^+^, Na^+^, H^+^, etc.). The addition of these salts to the mobile phase gives rise to the hydrophobic effect, which drives the hydrophobic cavities of the molecules onto the hydrophobic areas of the stationary phase [78]. Researchers have devised a dual-function stationary phase that exhibits cation exchange as well as hydrophobic interaction characteristics. This novel stationary phase has a porous silica gel which has been functionalized with both sulfonic and benzyl groups, making it capable of hydrophobic interactions at high salt concentrations and ionic interactions at low salt concentrations. Using this column, more than 96% of the mass and activity of the proteins were recovered [79].

Affinity chromatography was invented by Cuatrecasas and co-workers in 1968. It relies on the reversible binding between proteins and their cognate ligand, like antigen–antibody interaction. The interaction is highly specific and the target protein upon separation can be eluted out by denaturing agents or changing the pH or ionic strength. Pre-activated matrices are available for affinity chromatography, some of which are UltraLink Iodoacetyl resin, CarboLink Coupling resin, Profinity™ Epoxide resin and Affi-Gel 10 and 15 [80]. The importance of affinity chromatography has increased over the years owing to the application of recombinant DNA technology. DNA sequences that code for protein tags can be incorporated into the gene of interest, which will make the identification, purification and isolation of the target protein much easier. One of the most of popular affinity tags is the histidine tag, which is a sequence of six histidine residues at the end of the target protein sequence [81]. Most tags have the tendency to interfere with the folding of the protein or with its biological activity. To address this problem, fusion tags were developed, which increase expression yields and facilitate solubility and native folding. A few examples of fusion proteins include glutothoine s-transferase, maltose-binding protein, thioredoxin A, etc. [61].

#### 7.3.4. Electrophoresis

Electrophoresis separates proteins on the basis of net electric charge in macroporous gels or 1%–2% agarose. It is a very useful tool in protein separation, especially if the concentration of the protein fraction of interest happens to be too low. Gels such as polyacrylamide are used to separate protein on the basis of their molecular size. Proteins that are separated using this technique are eluted using proper blotting techniques. Electrophoresis has evolved into different types according to their purpose. For example, with the addition of mild detergent (such as sodium dodecyl sulphate, SDS), the electrophoresis gel enables us to analyze different subunits that constitute a protein molecule [82]. Performing electrophoresis without SDS will yield the native form of the protein. Isoelectric focusing is the separation of proteins based on their isoelectric point. This technique provides resolution of the target protein, but has some drawbacks. Proteins precipitate at their isoelectric point, which can contaminate other bands when using the Sephadex TM bed as the anticonvection medium in the experiment. This problem can be avoided by compartmentalizing the separation chamber [83].

#### 7.3.5. Expansion Bed Adsorption

Expansion bed adsorption for the purification of protein is a one-step method to remove cells and cell debris from large volumes of cellular homogenates. It is primarily a customized version of the fluidized bed reactor for protein separation. It involves a fluidized bed, where the adsorbent particles are subjected to an upward flow of liquid which keeps them suspended and separated. The cell and cell debris pass through the void present between the adsorbent particles. The total mixing in a fluidized bed reactor leads to the incomplete adsorption of target molecules onto the adsorbent particles. In a study involving this technique, β-galactosidase was purified in an expansion bed adsorption using Streamline-diethylaminoethanol (DEAE). Up to 65% of the total amount of enzyme was eluted, and, on analysis, the specific activity was found to have increased 12.6-fold [84].

#### 7.3.6. Membrane Adsorption

As the name suggests, this method utilizes a modified membrane to selectively adsorb protein molecules of interest. The advantage of this method over conventional column chromatography is that it circumvents the problems associated with diffusion. Convective flow through these membranes minimizes mass transport resistance, limiting it to film diffusion at the membrane matrix surface. This makes the adsorption–desorption cycle much easier, facilitating higher flow rates, thereby decreasing the operational span. Researchers at the Department of Chemistry, Michigan State University, demonstrated how high-capacity protein-adsorbing membranes can be created from poly (acrylic acid)-containing films by simply lowering the pH. Lowering the pH generated –COOH groups that form sites for ion exchange or for metal-ion complexes to get attached, which can selectively bind to target proteins. Subsequently, they were able to bind histidine6-tagged ubiquitin with >90% recovery from the cell extract [85].

### 7.4. One-Step Immobilization and Purification

Enzymes can be immobilized and purified in a single step, and techniques that aid this process are becoming more and more popular. In general terms, a single interaction between the enzyme and the support material is all it takes to stabilize the enzyme, and, at other times, several interactions may be necessary for the protein molecule to remain attached to the support. However, as is obvious, the focus of enzyme immobilization should be multi-point or multi-subunit attachment to improve enzyme stability [86]. Enzyme immobilization and purification via one step can be achieved by following three different strategies:

(i) immobilization via one point;
(ii) introduction of different domains; and
(iii) immobilization using heterofunctional supports.

Immobilization via one point employs custom-made supports that are specific to the target protein based on certain structural features by the formulation of heterofunctional supports to immobilize a specific enzyme via multipoint attachment and, the application of site-direct mutagenesis in the effort to introduce specific domains in the target protein molecule that show affinity to the heterofunctional supports. The immobilization of lipases on hydrophobic supports by means of interfacial activation is a special case.

An example of single point immobilization is the use of antigen–antibody interaction. This is a selective process with high levels of sensitivity, because only the target protein becomes immobilized. Monoclonal or polyclonal antibodies are used for this purpose. Two factors that govern the success of this procedure are (i) the immobilization of the target protein on the antibody and (ii) the prevention of any undesired adsorption. The use of monoclonal antibodies gives the user the flexibility to decide the orientation of the enzyme with respect to the support surface. This is an important feature since it permits the user to choose the orientation where the active site of the enzyme is fully exposed [87]. This may also help in safeguarding the enzyme if the site of attachment is a fragile region which can be inactivated by inhibitors. The immobilization yield following this procedure is 100% and an almost pure immobilized enzyme is achieved [88]. In some cases, the antibody is not specific to the target protein, but to a certain domain that has been introduced by site-directed mutagenesis. Using this step, the correct orientation of the target protein can be guaranteed. Also, any protein with the required domain can be immobilized on this support. In this case as well, the immobilization is almost 100%, with the achievement of total purity of the enzyme, but the stabilization is not that significant, since there is no interaction between the enzyme and the support material [89].

The second technique is by the introduction of different domains that induce affinity between the protein molecule and the support. These domains are peptides that are very small, e.g., cellulose-binding domain and other artificial peptides that are proteinaceous in nature like his-tag. In the case of lipases, it is possible to take advantage of specific catalytic mechanisms. Interfacial activation is a mechanism used by lipases to act on the surface of oil drops. Lipases have a very large hydrophobic active centre, which is protected by a lid which is a polypeptide chain, while exposing its hydrophilic exterior when in an aqueous environment. In this confirmation, the lipase is essentially closed. There is yet another form called the open form, where the hydrophobic catalytic centre is exposed; both forms shift between each other and are in equilibrium. So, in the presence of an oil drop, the lid is displaced exposing the hydrophobic catalytic core and the equilibrium taking up an open confirmation. This property of lipases where they can adsorb onto hydrophobic surfaces is called the interfacial activation [90,91,92].

Interfacial activation can be induced by the introduction of any form on hydrophobic substance, such as an oil drop or a hydrophobic support, or even a hydrophobic protein, for that matter. All these materials have been extensively used for the purification of lipases. The immobilization of lipases on a hydrophobic support at low ionic strength permits the stabilization of the open form on the enzyme in one single step and therefore is a much applied step for purification [93,94]. This process works only in low ionic strengths. High ionic strength during immobilization/purification favours the lipase to be in closed form and favours the adsorption of other proteins onto the substrate. The conventional adsorption process of lipase is not as efficient as interfacial activation [95]. Based on this criterion, lipases behave in different ways when it comes to adsorption on a hydrophobic support. Some lipases require highly hydrophobic supports, while others can only be adsorbed on mild ones. The nature of the group on the support can also be a determining factor for lipase purification. Usually, octyl agarose and sepabeadsdecaoctyl are supports that can contribute to lipase stability by 1000-fold [96]. This protocol is very simple and can easily be used on an industrial level. One of the most popular commercialized immobilized lipase preparations, Novozym 435, is prepared using this technique.

In some cases, immobilization of the protein by just one link between the enzyme and the support is desired, considering the risks involved, like desorption of some molecules, and to improve enzyme stability. In situations like these, heterofunctional groups are desired, with the tag interacting with one domain of the enzyme while other groups get involved in the formation of multipoint covalent bonds [97]. Heterofunctional supports are matrices with several functional groups on their surface, capable of physical and chemical interactions intended to interact with the protein of interest. Some of the groups present adsorbs the target protein, while the rest of the functionalities contribute to making the protein–support interaction irreversible. This strategy enables the purification of the protein by selective adsorption. This method is applicable to large proteins with multimeric subunits. Epoxides and glyoxyl groups are the chemically reactive groups used in this mode of immobilization/purification. Glyoxyl groups cannot immobilize proteins in neutral pH [98,99,100]. After adsorption, the enzyme may interact with the support groups to form covalent bonds contributing to an increase in stability. Increasing the pH can result in an increase in the reactivity of nucleophilic groups on the surface of the enzyme onto the support [101].

## 8. Technical Problems

Low enzyme yield, low enzyme titre and low enzyme productivity are all mainly caused due to technical problems that prevent microorganisms to thrive on lignocellulosic hydrolysates. Some of these technical problems include the inability to control the C:N ratio due to the addition of nitrogen rich cellulases and hemicellulases, low sugar concentrations and the presence of 5-C and 6-C sugars, causing diauxic growth. However, the major issue associated with the use of lignocellulose for fermentation is the presence of microbial inhibitors: furfural, hydroxymethyl furfural, acetic acid, phenolic compounds and other chemical species. These compounds are a by-product of lignin breakdown during harsh pretreatments. The mechanism of how phenolic compounds inhibit growth is not understood completely. Vanillin, syringaldehyde, trans-cinnamic acid, and hydroxybenzoic acid are some of the compounds that inhibit enzyme activity [102].

The major methods that are used to eliminate the ill effects of inhibitors include (i) preventing the formation of inhibitors during pretreatment and hydrolysis, (ii) hydrolysate detoxification, (ii) development of microorganisms capable of tolerating them and (iv) neutralizing the toxic compounds. The formation of toxic compounds can be prevented by careful selection of the lignocellulosic material and the application of mild pretreatments. Certain chemicals can be used to detoxify the hydrolysate. Over-liming utilizes Ca(OH)_2_ for lignocellulose pretreatment and is one of the most economical processes for detoxification [103]. Alriksson, Horvath [104] used NH_4_OH as a source of nitrogen as well as a measure against inhibitors. At pH 10.0, the addition of NH_4_OH resulted in a substantial removal of furfural and hydroxymethyl furfural concentrations, increasing the fermentation efficiency, as well. Enzyme immobilization is also an effective measure to tackle inhibitors [105].

Enzymes have been used for the removal of toxic compounds. Laccase and peroxidase enzymes obtained from *Trametes versicolor* have been effective in removing phenolic compounds from hemicellulose hydrolysate [106]. Certain microorganisms have the capability to metabolize enzyme inhibitors. *Coniochaeta ligniaria* NRRL30616, a fungal strain, is capable of metabolizing furfural, HMF and phenolic derivatives. *C. ligniaria* NRRL30616 was able to convert the toxic molecules either into biomass or into less harmful chemicals. Furthermore, a higher cellulose conversion rate was observed [107]. Vacuum evaporation is one of the most common physical methods used to remove inhibitors. It results in the complete removal of volatile fractions, such as acetic acid, furfural and vanillin. The drawback of this method is the concentration of non-volatile compounds lignin derivatives. Extraction can also be used to remove harmful phenolic compounds. Kim, Ximenes [108], found that phenolics present in the lignocellulose hydrolysate was responsible for reducing enzyme activity. Accordingly, upon removal of the same, complete enzyme activity was regained. The phenolic compounds can be removed by PEG surfactant, activated charcoal and/or ethyl acetate.

## 9. Conclusions

Lignocellulosic food industry waste is the cheapest and most opulent of carbohydrates available for valorization and subsequent value addition. Fungal organisms show significantly lower yields when using a low carbohydrate substrate, such as lignocellulose. This is due to the heterogeneous nature of cheap carbon sources, because microorganisms have different uptake rates for 5-C and 6-C sugars. Pretreatments that enable a higher rate of utilization of lignocellulosic biomass, as well as high saccharification rates at where hydrolyzing enzymes are employed, can improve the economics of enzyme production. Solid state fermentation triumphs over the submerged fermentation method in maintaining low cost of production. Lignocellulosic food waste shows a lot of promise and can be utilized as a carbon source in mainstream, upscale fermentation processes.

## Figures and Tables

**Table 1:** Summary of the lignocellulosic sources that have been used as raw material for enzyme production.

Enzyme	Feedstock	Bioprocessing Conditions	Microbial Strain	Application	Reference
Amylase	Brewers’ spent grain hydrolysate	Submerged fermentation mode, hydrolysate used as additive in amylase production media, fermentation time: 30 h	Catabolite-repressed *Bacillus subtilis* KCC103	Baking, brewing, animal nutrition, aquaculture, biofuel, dishwashing and laundry detergents	[58]
β-glucanase	Oatmeal Orange peel	Solid state fermentation mode, 50% w/w moisture, temperature: 30 °C, pH: 5.5, fermentation time: 4 days Solid state fermentation mode, orange peel with basal media, incubated at 30 °C, pH: 5.5, fermentation time: 4 days	*Rhizomucor miehei* CAU432 *Tricoderma viride* MBL	Brewing, bioethanol	[109,110,111]
Cellulase	Apple pomace Banana peel	Solid state fermentation mode, 75% initial moisture content, initial temperature: 30 °C, fermentation time: 48–72 h Solid state fermentation mode, 50% moisture content, temperature: 30 °C, fermentation time: 192 h	*Aspergillus niger* NRRL-567 *Trichoderma viride* GIM 3.0010	Detergents, bleaching, deinking, refining, starch modification, drainage improvement, decolourization of dyes in effluent, cellulosic and starch based ethanol, biodiesel	[112,113]
Inulinase	Yacon juice Banana peel, wheat bran, rice bran, orange peel, bagasse Soy bean cake	Submerged batch fermentation mode, temperature: 30 °C, pH: 5, fermentation time: 7 days Solid State fermentation mode, 65% moisture content, temperature: 35 °C, fermentation time: 72 h Solid state fermentation mode, 60% moisture content, K_2_HPO_4_, ZnSO_4_·7H_2_O, temperature: 30 °C, fermentation time: 48 h	*Aspergillus kawachii* *Saccharomyces* sp. *Pencillium rugulosum* (MTCC-3487)	Production of high-fructose corn syrup	[32,114,115,116]
Invertase	Red carrot jam processing residue Sugarcane bagasse Orange peel, pineapple peel waste	Solid state fermentation mode, temperature: 30 °C, fermentation time: 72 h Solid state fermentation mode, temperature: 30 °C, fermentation time: 72 h Solid state fermentation mode, temperature: 30 °C, fermentation time: 32 h, initial pH: 5.5	*S. cerevisiae* NRRL Y-12632 *Aspergillus niger* GH1, *Cladosporium cladosporioides*	Sucrose hydrolysis	[117,118,119]
Lactase	Fermented ragi	Submerged fermentation mode, media supplemented by 0.75% lactose and 1% ragi, fermentation time 12 h, pH 5.5	*Lactobacillus acidophilus*	Dairy, preparation of lactose-free food products	[120]
Mannanase	Apple pomace and cotton seed powder mixture	Solid state fermentation mode, 50% initial moisture content, pH 5.5, temperature 30 °C, fermentation time 48 h	*Aspergillus niger* SN-09	Paper and pulp, textile, pharmaceuticals	[121,122,123]
	Palm kernel cake	Solid state fermentation mode in stainless steel horizontal bioreactor, initial moisture content 1:0.75 (*w*/*v*), temperature 30 °C, fermentation time 4 days.	*Aspergillus terreus* SUK-1		
	Passion fruit peel	Submerged fermentation mode, pH 6.5, 8.6 days	*Pencillium verruculosum*		
Pectinase	Orange peel	Submerged fermentation mode, temperature 35 °C, pH 5.2, fermentation time: 3 days	*Pencillium oxalicum* PJ02 *Aspergillus niger*	Processing of starch and wine, juice processing	[124,125]
	Deseeded sunflower head	Solid state fermentation mode, temperature: 34 °C, initial moisture content: 65%, fermentation time: 120 h Submerged fermentation mode, temperature: 34 °C, pH: 5.0, fermentation time: 120 h			
Xylanase	Coffee by-products	Solid state fermentation mode, initial moisture content: 50%, temperature: 30 °C, fermentation time: 5 days.	*Pencillium* sp. CFR 303	Bleaching and deinking of paper, baking, animal nutrition	[126]
Protease	Brewer’s spent grain, corn steep liqour	Submerged fermentation mode, temperature: 28 °C, fermentation time: 6 days	*Streptomyces malaysiensis* AMT-3,	Food, pharmaceutical, animal feed, leather, diagnostics, waste management	[127]
Transglutaminase	Industrial fibrous soy residue	Solid state fermentation mode, temperature: 33 °C, fermentation time: 48 h	*Bacillus circulans* BL32	Meat processing, dairy products, baking, edible film, leather finishing, cosmetics	[128]
Laccase	Wheat bran	Stirred bioreactor working volume: 120 L, temperature: 30 °C, pH: 6.0, fermentation time: 4 days	*Cerrena unicolor* C-139	Bleaching, deinking of paper, polishing and preparation of textiles	[129]
Lipase	Banana peel, potato peel, cassava peel	Solid state fermentation mode, initial moisture content: 55%, temperature: 30.5 °C, fermentation time: 60 h	*Aspergillus niger*	Meat processing, detergents, degreasing, dehairing of leather	[130,131]
Phytase	Orange and citrus peel	Solid state fermentation mode, temperature: 50 °C, fermentation time: 72 h	*Klebsiella* sp. DB-3FJ711774.1	Animal nutrition	[132]
Polygalacturonase	Orange peel, wheat bran	Solid state fermentation mode, temperature: 22.4 °C to 27.5 °C, incubation period: 3.8 to 5.5 days	*Aspergillus sojae* M3	Catalyzes the hydrolysis of α-1,4-glycosidic linkages in pectic acid. Used in food industry.	[133,134]
Cellulase	Pea pod waste	Solid state fermentation mode, moisture content of 70% made up by Mendel Weber medium, temperature: 30 °C, fermentation time: 96 h	*Aspergillus niger* HN-1	Detergents, bleaching, deinking, refining, starch modification, drainage improvement, decolourization of dyes in effluent, cellulosic and starch based ethanol, biodiesel	[135]
β-Xylosidase	Wet disc milling rice straw (WDMRS)	Submerged fermentation mode, working volume: 1 L in 2 L bioreactor, pH: 4.8, media composition: 20 g WDMRS, 5 g polypeptone, 4 g urea, 2 g (NH_4_)_2_SO_4_, 5 g (NH_4_)_2_HPO_4_, 5 g KNO_3_, 15 g KH_2_PO_4_, 1 g Tween 80, fermentation time: 96 h	*Trichoderma asperellum* KIF125	Baking, improving digestibility of animal feed, production of d-xylose for xylitol manufacture, deinking of recycled paper	[136]
β-fructofuranosidase (invertase)	Apple pomace	Submerged fermentation mode, working volume: 25 mL in 125 mL Erlenmeyer flask, initial pH: 7.5, fermentation time: 12 days, room temperature	*Aspergillus versicolor*	Food additive	[137]
